# Concurrent Detection of Circulating Minor Histocompatibility Antigen-Specific CD8^+^ T Cells in SCT Recipients by Combinatorial Encoding MHC Multimers

**DOI:** 10.1371/journal.pone.0021266

**Published:** 2011-06-24

**Authors:** Kelly Broen, Annelies Greupink-Draaisma, Rob Woestenenk, Nicolaas Schaap, Anthony G. Brickner, Harry Dolstra

**Affiliations:** 1 Laboratory of Hematology, Department of Laboratory Medicine, Radboud University Nijmegen Medical Centre, The Netherlands; 2 Department of Hematology, Radboud University Nijmegen Medical Centre, The Netherlands; 3 Departments of Medicine and Immunology, University of Pittsburgh Cancer Institute, Pittsburgh, Pennsylvania, United States of America; University of Montreal, Canada

## Abstract

Allogeneic stem cell transplantation (SCT) is a potentially curative treatment for patients with hematologic malignancies. Its therapeutic effect is largely dependent on recognition of minor histocompatibility antigens (MiHA) by donor-derived CD8^+^ T cells. Therefore, monitoring of multiple MiHA-specific CD8^+^ T cell responses may prove to be valuable for evaluating the efficacy of allogeneic SCT. In this study, we investigated the use of the combinatorial encoding MHC multimer technique to simultaneously detect MiHA-specific CD8^+^ T cells in peripheral blood of SCT recipients. Feasibility of this approach was demonstrated by applying dual-color encoding MHC multimers for a set of 10 known MiHA. Interestingly, single staining using a fluorochrome- and Qdot-based five-color combination showed comparable results to dual-color staining for most MiHA-specific CD8^+^ T cell responses. In addition, we determined the potential value of combinatorial encoding MHC multimers in MiHA identification. Therefore, a set of 75 candidate MiHA peptides was predicted from polymorphic genes with a hematopoietic expression profile and further selected for high and intermediate binding affinity for HLA-A2. Screening of a large cohort of SCT recipients resulted in the detection of dual-color encoded CD8^+^ T cells following MHC multimer-based T cell enrichment and short *ex vivo* expansion. Interestingly, candidate MiHA-specific CD8^+^ T cell responses for LAG3 and TLR10 derived polymorphic peptides could be confirmed by genotyping of the respective SNPs. These findings demonstrate the potency of the combinatorial MHC multimer approach in the monitoring of CD8^+^ T cell responses to known and potential MiHA in limited amounts of peripheral blood from allogeneic SCT recipients.

## Introduction

In HLA-identical allogeneic stem cell transplantation (SCT), alloreactive CD8^+^ T cells specific for minor histocompatibility antigens (MiHA) play a pivotal role in graft rejection, graft-versus-host disease (GVHD) and the curative graft-versus-tumor (GVT) response. Several MiHA have been molecularly defined with the potential to induce a GVT response without inducing GVHD, such as HA-1 [1]–[4], LRH-1 [5] and ACC-1 [6]. Although MiHA can be regarded as the most dominant antigens in GVT immunity, the CD8^+^ T cell response rate towards these antigens has not been followed extensively in transplanted patients. Furthermore, most analysis focused on the detection of CD8^+^ T cell responses to single MiHA epitopes using conventional techniques such as single-tetramer staining or the ELISPOT assay.

Fluorescent labeled peptide-major histocompatibility antigen (MHC) complexes, known as MHC multimers, are excellent reagents to monitor MiHA-specific T cell responses after SCT and donor lymphocyte infusion (DLI) in peripheral blood of transplanted patients. Especially, the recently developed combinatorial encoding technique using dual-color encoded MHC multimers is a very attractive approach to accurately detect multiple MiHA-specific T cells in one sample [7]. The principle of this method relies on the flow cytometric detection of a single T cell population that is stained with different fluorochrome-labeled MHC multimers. This dual-color encoded MHC multimer approach has the ability to detect up to 15 different T cell populations when using 6 different fluorochromes [7]. Therefore, a key advantage compared to single-tetramer staining is that the amount of patient peripheral blood cells needed is equal to just one labeling, making the technique very suitable when dealing with limited amounts of patient material. The combinatorial encoding approach can accommodate a wide range of different peptide-MHC multimers for several HLA molecules, which can be readily produced through UV-mediated ligand exchange [8], [9]. The versatility of these two methods makes the combinatorial encoding MHC multimer technique an excellent monitoring tool for detecting MiHA-specific CD8^+^ T cell responses against a panel of known MiHA.

Furthermore, another potential application of the combinatorial encoding MHC multimer approach could be its use to identify new MiHA. Recently, the value of the method for antigen discovery has been demonstrated for the identification of melanoma-associated T cell epitopes [7]. Here, we explored the use of the combinatorial MHC multimer technique for the detection of CD8^+^ T cell responses in transplanted patients against candidate MiHA defined through a reverse immunology approach. Interestingly, we detected peptide-specific dual-tetramer positive CD8^+^ T cells against 8 out of 75 HLA-A2 binding peptides that were predicted *in silico* from polymorphic hematopoietic-specific genes.

Collectively, our results illustrate that the combinatorial MHC multimer method is a suitable technique to analyze patients after SCT and DLI for the concurrent occurrence of MiHA-specific CD8^+^ T cells targeting known MiHA or candidate MiHA identified by reverse immunology approaches.

## Results

### Immunomonitoring of MiHA-specific CD8^+^ T cell responses using combinatorial MHC multimer staining

The success rate of immunological responses in patients post-SCT and DLI can be assessed by measuring the MiHA-specific T cells present in the blood of the patient [1], [2], [5]. Since patient material is often in short supply, we developed a technique that can screen for multiple MiHA-specific T cells in a limited amount of patient follow-up material. This technique makes use of dual-color-encoded MHC multimers to detect multiple antigen-specific T cells in one sample [7], [10]. In order to set up such a MiHA-multimer kit, we have chosen a set of 10 MiHA, each being encoded by two different fluorochromes coupled to an MHC multimer containing an individual peptide (Table 1).

**Table 1:** MHC multimers and combinatorial encoding scheme as assembled in MiHA-multimer kit.

MiHA	HLA	Peptide sequence	Reference	Fluorochrome code	
SMCY	A2	FIDSYICQV	[29]	PE	APC
HY	B7	SPSVDKARAEL	[30]	PE	Qdot605
HA-1	A2	VLHDDLLEA	[3]	PE	Qdot655
LRH-1	B7	TPNQRQNVC	[5]	PE	Qdot705
ADIR	A2	SVAPALALAFPA	[31]	APC	Qdot605
ACC-1	A24	DYLQYVLQI	[6]	APC	Qdot655
HA-8	A2	RTLDKVLEV	[32]	APC	Qdot705
SP110	A3	SLPRGTSTPK	[22]	Qdot605	Qdot655
PANE-1	A3	RVWDLPGVLK	[18]	Qdot605	Qdot705
HA-2	A2	YIGEVLVSV	[33], [34]	Qdot655	Qdot705

**Figure 1:**
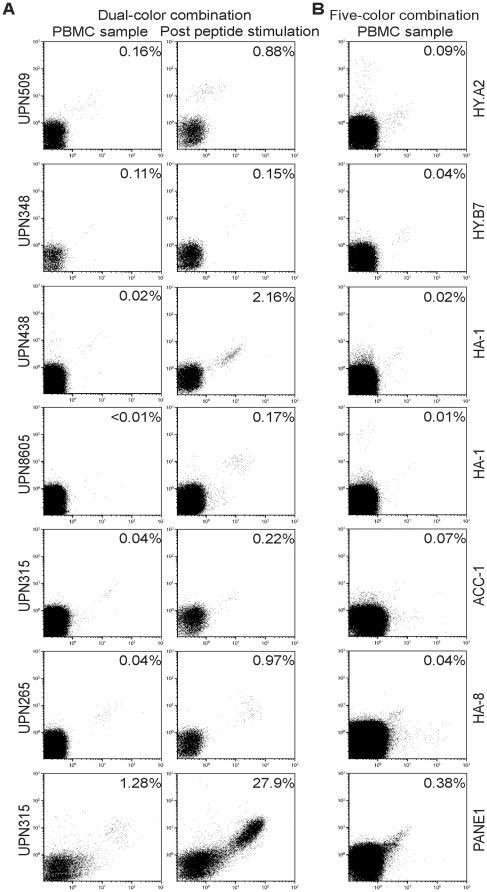
Detection of MiHA-specific CD8^+^ T cells in peripheral blood of patients after SCT and DLI. (**A**) PBMC collected post SCT stained with PE- and APC-conjugated tetramers, CD8 AlexaFluor 700, CD4-, CD14-, CD16- and CD19-FITC and Sytox Blue. The remaining PBMCs were stimulated once with the MiHA peptide pulsed (10 µM) EBV-LCL of the donor and assayed on day 7 for tetramer^+^ CD8^+^ T cells. (**B**) PBMC stained with combinatorial encoded MiHA multimers, CD8 AlexaFluor 700, CD4-, CD14-, CD16- and CD19-FITC and Sytox Blue. Subsequently, cell populations were analyzed on the Cyan cytometer and re-analyzed by Kaluza 1.1. Cells were gated on CD8^+^FITC^−^Sytox Blue^−^ lymphocytes, and the percentage of tetramer^+^ cells among CD8^+^ T cells is depicted.

To determine whether the developed MiHA-multimer kit is suitable for the detection of multiple MiHA-specific T cells in blood samples of transplanted patients, we selected a cohort of patients transplanted with partially T-cell depleted stem cell grafts from HLA- identical sibling donors. These patients also received pre-emptive DLI if they did not develop significant GVHD after SCT. These patients and their respective donor were typed for MiHA mismatches using fluorescence-based competitive allele specific PCR (Table 2). The selected SCT recipients were mismatched for one or two MiHA with their HLA-identical donor and no tetramer^+^ T cells were observed in pre-SCT recipient PBMC (Table 2). For the disparate MiHA a dual-color tetramer analysis was performed using both APC and PE labeled tetramers (Figure 1A). Since double positive events were scored, the dual-color tetramer stainings result in accurate detection of low frequency MiHA-specific CD8^+^ T cells [10], even at relatively late time points post-SCT. Specific detection of these low frequency MiHA-specific-CD8^+^ T cell populations was verified after *in vitro* stimulation of peripheral blood mononuclear cells (PBMC) samples with MiHA peptide-pulsed EBV-LCL (Figure 1A).

**Table 2:** Characteristics of SCT recipients containing circulating MiHA-specific CD8^+^ T cells.

UPN	Diagnosis	Sex (Rt/Do)	HLA-A		HLA-B		DNA MiHA mismatch	Tetramer^+^ cells by dual colorPre-SCT	Screened material - Time post SCT (wks)	Tetramer^+^ cellsDual color vs five color*	
509	NHL	M/F	1	2	8	51	HA-8	<0.01%	30 post SCT	<0.01%	<0.01%
							HY	<0.01%	30 post SCT	0.16%	0.09%
348	CML	M/F	1	11	7	8	HY	<0.01%	31 post SCT	0.11%	0.04%
438	NHL	F/M	2	31	51		HA-1	No material	62 post SCT	0.02%	0.02%
8605	MM	F/M	2	11	51	35	HA-1	<0.01%	74 post SCT	<0.01%	0.01%
315	AML	M/M	3	24	7	35	ACC-1	<0.01%	68 post SCT	0.04%	0.07%
							PANE1	<0.01%	68 post SCT	1.28%	0.38%
265	AML	M/F	2	3	38	47	HA-8	No material	24 post SCT	0.04%	0.04%
							HY	No material	24 post SCT	<0.01%	<0.01%

*Statistical analysis between dual-color and five-color tetramer analysis was performed using the unpaired t test. The p value *is* 0.2903.

Abbreviations: Rt, recipient; Do, donor; AML, acute myeloid leukemia; CML, chronic myeloid leukemia; NHL, non-Hodgkin lymphoma; MM, multiple myeloma.

**Figure 2:**
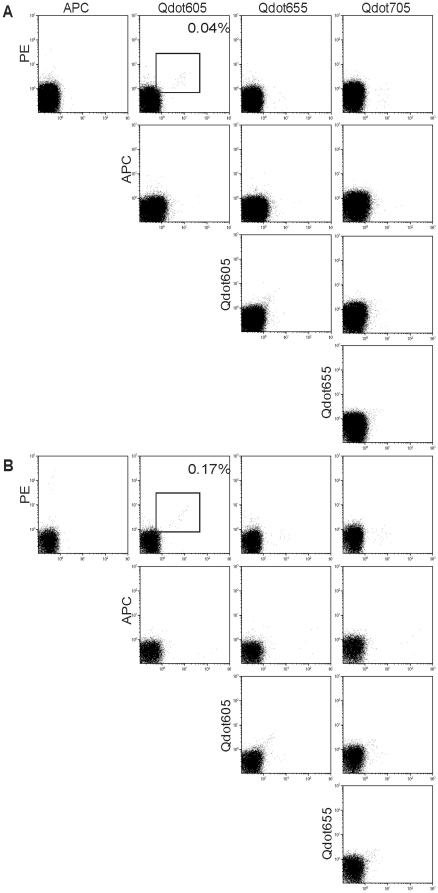
Detection of peptide-specific T cells by the combinatorial encoding MiHA-multimer assay. (**A**) Ten different flow cytometric dot plots showing thawed PBMC of representative patient UPN348, stained with the combinatorial encoded MiHA-multimer assay using the peptide set as described in Table 1. The combination of multimers coupled to PE and to Qdot605 shows a positive response, corresponding to multimer-SMCY.A2^+^ T cells. Cells were stained with MiHA multimers, CD8 AlexaFluor 700, CD4-, CD14-, CD16- and CD19-FITC and Sytox Blue. Subsequently, cell populations were analyzed by flow cytometry. Cells were gated on CD8^+^FITC^−^Sytox Blue^−^ lymphocytes and corrected for single, triple or quadruple multimer-positive cells. The percentage of multimer^+^CD8^+^ T cells is depicted. (**B**) Ten different dot plots showing PBMC of representative patient UPN348 after culturing with SMCY.A2-peptide pulsed donor EBV-LCL. Only the response observed in non-cultured PBMC show an increase of multimer-SMCY.A2^+^ T cells. Staining and analysis was performed as described under (A).

Knowing the MiHA-specific T cell status of the patient samples, we tested a second cryopreserved PBMC sample again by the new five-color combinatorial encoding MiHA-multimer kit (Figure 1B). Results of a representative patient (i.e. UPN348) can be seen in Figure 2A. For this patient's cryopreserved PBMC sample at 31 weeks post-SCT, we observed 0.11% HY.B7-tetramer^+^ cells using dual-color staining (Figure 1A), compared to 0.04% by the five-color MiHA-multimer kit (Figure 1B and 2A). Specific detection of CD8^+^tetramer^+^ T cells was verified after *in vitro* stimulation of PBMC samples with HY-B7 peptide-pulsed EBV-LCL, showing 0.17% HY-B7-tetramer^+^ cells with the MHC-multimer kit (Figure 2B) compared to 0.15% by dual-color staining (Figure 1A). All different MiHA-specific CD8^+^ T cells found by dual-color staining could also be found in the thawed PBMC samples analyzed by the new five-color combinatorial encoded MiHA-multimer assay, although the frequency of MiHA-specific cells was somewhat lower (Table 2). These data demonstrate that circulating MiHA-specific CD8^+^ T cells can be detected concurrently using a combinatorial encoding MiHA-multimer kit, making this a valuable tool for immunomonitoring of SCT recipients using a limited amount of patient material.

### Identification of novel candidate MiHA by reverse immunology

Next, we studied the feasibility of the combinatorial encoding MHC-multimers as an identification tool for the discovery of novel MiHA. In order to predict HLA-A2-restricted candidate MiHA within hematopoietically restricted gene products, we developed and tested an adaptable computer algorithm. An arbitrary minimum predicted halftime of dissociation (T_(½)_) of 30 seconds was set for candidate HLA-A2 restricted MiHA, based on corroboration with known HLA-A2 peptide and MiHA binding affinities, which indicated that a peptide with moderate HLA-A2 binding affinity would exhibit a predicted T_(½)_ of >30 seconds. Subsequently, candidate MiHA were further selected, based upon either observed allele frequencies in Caucasians or allele frequencies reported by dbSNP/HapMap. Typically, only those candidate MiHA that had a reported allele frequency for the candidate MiHA-positive allele between 15% and 46% were pursued as candidate MiHA, since MiHA with frequencies within this range yield a theoretical clinical applicability of more than 20% in HLA-A2 matched unrelated recipient-donor pairs.

**Figure 3:**
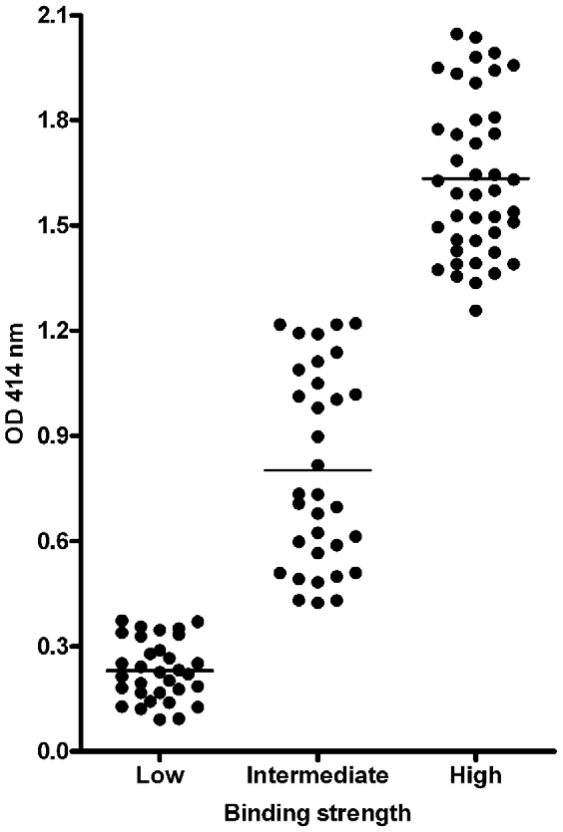
ELISA-based HLA-A2 affinity assay for 107 *in silico* predicted potential MiHA. HLA-A2 monomers containing UV degradable peptide were exchanged with 107 predicted MiHA peptides and HLA-A2 binding affinity was tested. Results show 32 low binders (OD≤0.39), 33 intermediate binders (OD 0.4–0.124) and 42 high binders (OD≥1.25). Data are depicted as mean of triplicate wells.

Peptides of the predicted candidate MiHA were synthesized and HLA-A2 monomers were generated using the UV-mediated ligand exchange method [9]. All monomers were tested for stable binding of the predicted epitope using an ELISA-based HLA-affinity assay [9], which revealed that 75 out of 107 predicted potential MiHA bind HLA-A2 with high (OD≥1.25) or intermediate (OD 0.4–1.24) affinity (Figure 3). As a positive control for successful peptide exchange, viral (EBV, CMV, FLU) HLA-A2 specific peptides were used, as well as negative controls of not applying UV light or not adding any peptide at all (data not shown). Peptides with low affinity to HLA-A2 (OD≤0.4) were discarded from further experiments and only MHC-monomers harboring peptides that bound with high or intermediate strength were used for multimerization. This set of MHC multimers was color coded, resulting in 5 pools of 15 different MHC multimers and 1 pool of 4 different MHC multimers (Supplemental Table S1). In addition to the predicted potential MiHA, pool A-D contained control MHC multimers for the MiHA HA-1.A2, HA-2.A2, HA-8.A2 and SMCY.A2.

### Concurrent detection of multiple peptide-specific CD8^+^ T cells after enrichment and expansion

**Figure 4:**
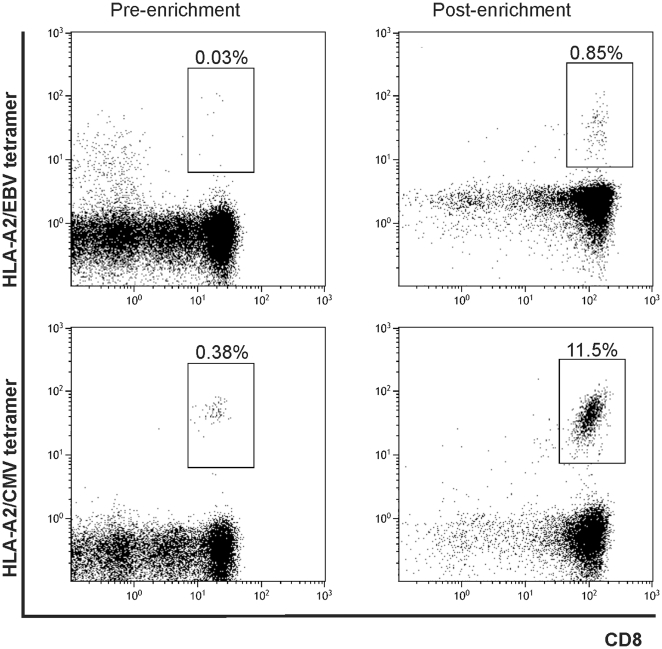
PBMC of a healthy donor enriched for virus-specific T cells. Peripheral blood of an HLA-A2^+^ healthy donor was enriched for EBV- and CMV-specific CD8^+^ T cells. Before culture there were low amounts of EBV- and CMV-specific CD8^+^ T cells present, which showed an increase after enrichment and 16 days of culture. Cells were stained with tetramers coupled to PE and gated on CD8^+^tetramer^+^. Analysis was performed on the Cyan cytometer.

To investigate whether peptide-specific CD8^+^ T cells could be found for the predicted MiHA epitopes that had high or intermediate affinity to HLA-A2, a cohort of 31 patients was selected for screening of the follow-up material collected after DLI. Approximately half of the patients selected, developed acute GVHD after SCT and/or acute or chronic GVHD after DLI. These observations show the presence of an allo-response that might have been mediated by MiHA-specific T cells making these patients attractive to look for candidate MiHA. To detect epitope-specific-CD8^+^ T cells in the patient PBMC collected after DLI (average 18 weeks post DLI) we first enriched the samples using PE-labeled MHC multimers. Figure 4 shows an example of an enriched culture containing low frequency CD8^+^ T cells specific for the HLA-A2-restricted EBV (GLCTLVAML) and CMV (NLVPMVATV) epitopes (respectively 0.03% and 0.38%) pre-culture. MHC multimer-based enrichment and subsequent expansion yielded a CD8^+^ T cell population consisting of 0.85% EBV-TET^+^ and 11.5% CMV-TET^+^ cells after 16 days of culturing. This shows the potential of this method to increase low frequency T cells to sufficient cell numbers of multiple specificities for further experiments.

**Figure 5:**
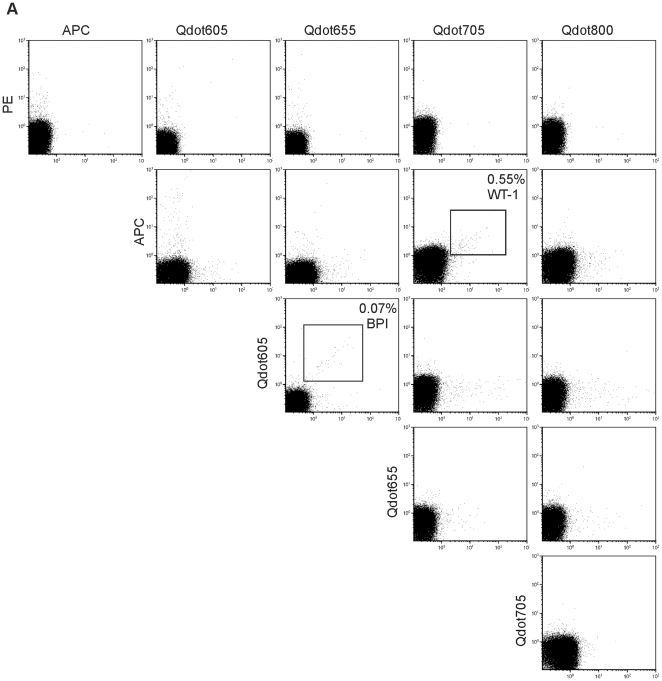
Detection of multiple peptide-specific CD8^+^ T cells after enrichment of patient PBMC. Fifteen different dot plots showing multimer-enriched PBMC of representative patient UPN665 stained with the combinatorial encoded MiHA assay using the multimers of pool A. Epitope-specific T cells were detected for RMFPNAPYL (APC and Qdot705) and KLQPYFQTL (Qdot605 and Qdot655). In addition to double positive cells, single positive cells for either APC, Qdot605, Qdot655 or Qdot705 are visible in their respective channels. Cells were stained with multimers, CD8 AlexaFluor 700, CD4-, CD14-, CD16- and CD19-FITC and Sytox Blue. Subsequently, cell populations were analyzed by flow cytometry. Cells were gated on CD8^+^FITC^−^Sytox Blue^−^ lymphocytes and corrected for single, triple, quadruple or quintuple multimer positive cells. The percentage of tetramer^+^CD8^+^ T cells is depicted in the dot plots.

To detect multiple MiHA peptide-specific CD8^+^ T cells against our pre-selected panel of 75 candidate HLA-A2-restricted peptides, we performed enrichments on 31 different patient samples of which 17 resulted in sufficient cell numbers for analysis. We detected peptide-specific CD8^+^ T cells in cultures of 5 different patients (Table 3). Notably, we detected a CD8^+^ T cell response against the known MiHA SMCY.A2. Additional patients were transplanted with donor grafts mismatched for any of the control HLA-A2 MiHA included in the analysis as was determined by SNP genotyping. However, we did not observe specific T-cell responses for these MiHA. Results of a representative patient (UPN665) are shown in Figure 5A. PBMC from this patient obtained at 14 weeks post DLI were enriched, expanded and analyzed for all 6 combinatorial MHC-multimer encoding pools. In pool A, two specific T cell populations were found. The combination of multimers coupled to APC and Qdot705 showed a positive response of 0.55% dual-color tetramer positive T cells, recognizing the WT-1-derived epitope RMFPNAPYL. In addition, the combination of multimers coupled to Qdot605 and Qdot655 showed a positive response of 0.07% dual-color tetramer positive T cells, recognizing the BPI-derived epitope KLQPYFQTL.

**Table 3:** Percentage of dual-color tetramer^+^CD8^+^ T cells in PBMC samples of transplanted patients screened after MHC-multimer based enrichment and short-term expansion.

Gene	Candidate MiHA	UPN466	UPN541	UPN665	UPN407	UPN427
WT-1	RMFPNAPYL			**0.55** a		
BPI	KLQPYFQTL	0.28	0.09	0.07	0.04	
LAG3	LTLGVLSLL			**0.19** b		
IGLL1	LMNDFYLGI					0.10
GPR109b	FLLEFLLPL			0.23	0.04	
CCRL2	ILATLPEFV			0.07		
CARD8	YLVPSDALL				**0.01** b	
TLR10	IQLPHLKTL				0.47	
SMCY.A2	FIDSYICQV				**0.07** b	

a Known WT-1 epitope.

b T cell response confirmed by SNP genotyping.

To verify whether the observed T cell responses correlate with SNP mismatches at the DNA level, we performed allele specific genotyping of donor and recipient for the SNPs corresponding to the immunogenic epitopes found in the combinatorial MHC-multimer staining. Although several mismatched SNPs between donor and recipient were found, only 2 out of 8 epitopes found in the screening corresponded with their respective SNP genotype (underlined in Table 3). These two candidate MiHA (i.e. LAG3 derived LTLGVLSLL and CARD100 derived YLVPSDALL), as well as epitopes to which a high response was observed (epitopes derived from WT-1 and TLR10), were selected for further experiments. However, dual-color tetramer stainings using PE- and APC-conjugated tetramers could not confirm specific CD8^+^ T cells for the found peptides in non-cultured PBMC. Although we were not able to verify responses found in the analysis, the use of combinatorial encoding MHC multimer panels proved useful for high throughput concurrent detection of specific CD8^+^ T cells against a large panel of candidate MiHA peptides.

## Discussion

The monitoring of multiple MiHA-specific T cells *in vivo* can give important additional information on the clinical response and might lead to more insight into the therapeutic applicability of specific MiHA. In this study, we report a novel approach for the monitoring of MiHA-specific T cells in a limited number of PBMC from patients after SCT and DLI, which we believe is of great value for the immunomonitoring of SCT recipients. Cryopreserved PBMC samples from patients who underwent partially T cell depleted allogeneic SCT, in some cases followed by DLI, were simultaneously analyzed for 10 different MiHA-specific T cells. In all 6 patients analyzed with the set of combinatorial encoded MHC multimers, MiHA-specific T cells could be detected. These MiHA-specific T cells proliferated upon stimulation with MiHA-peptide, showing a peptide-dependent response. To confirm functionality of the MiHA-specific T cells, proliferation upon stimulation with endogenously processed peptides should be measured. The specificity of the cells found in the analysis corresponded to the results obtained by conventional T cell screenings. The use of a combinatorial MiHA-multimer kit gives a quick overall view of numerous MiHA-specific T cells within patient blood samples post SCT. Moreover the amount of patient material used is equal to what conventional screenings use for just one specific staining. This method already has been applied as a tool for the simultaneous detection of autoreactive CD8^+^ T cells in blood [11] and now we show that it is a valuable tool for the concurrent detection of multiple MiHA-specific T cells after allogeneic SCT and DLI. The MiHA-detection kit used in this study already covers 10 prevalent MiHA, though flexibility of the technique allows further expansion of the MiHA set. However, the current technical limitations regarding the synthesis of HLA-monomers prevent implementation of certain MiHA, such as MiHA presented in HLA-B44. The MiHA-detection kit can be expanded by multimers coupled to additional fluorophores such as Qdot800 or Qdot565 and Qdot585. It should be noted that these three fluorophores have a low intensity for which should be corrected when combining with other fluorophores [7].

Because of the techniques' potential to screen for large numbers of different specific T cells [11] and its success in the identification of melanoma-associated T cell epitopes [7], we tested the potential of the combinatorial encoding approach to identify new MiHA that were predicted from a set of polymorphic genes expressed by the hematopoietic system. For the development of adjuvant tumor-specific immunotherapy after SCT it is important to enlarge the spectrum of molecularly identified MiHA that are selectively expressed in the hematopoietic system and in hematologic malignancies. Investing in new high throughput methods for MiHA identification is in our opinion critically important. The recently developed technique using dual-color-encoded MHC multimers to detect multiple antigen specific T cells in one sample [7] was tested for this purpose. The combinatorial encoding approach already led to the discovery of a new melanoma-associated epitope in a screening of T cells [7], showing its applicability in these kind of studies. Our present article describes the prediction of MiHA from genes with a hematopoietic restricted expression profile and the verification of these MiHA by screening for MiHA specific CD8^+^ T cells in patient PBMC following MHC multimer-based enrichment and a short-term expansion. A similar reverse immunology approach of predicting MiHA has already yielded a set of novel MiHA originating from the Y chromosome presented in HLA-A2 [12]. These male-specific MiHA were verified by means of ELISPOT showing some of these MiHA to be even more immunogenic than the well known SMCY.A2 male specific MiHA. Although this indicates that predicting MiHA can yield actual functional MiHA, this set of MiHA is not of therapeutic interest since it is not to be expected that these MiHA have a hematopoietically restricted expression profile.

Here we performed an analysis using combinatorial encoded multimers specific for 75 different epitopes. Of the 31 enriched cultures we screened 17 and found multimer-positive T cells in 5 of the cultures. One of the specific T cell populations found was directed against the tumor associated antigen WT-1 for which specific T cells can be found in CML [13], MDS and AML patients [14], [15]. In the set of predicted candidate MiHA, the gene WT-1 was integrated because of the presence of SNP rs9332973 resulting in the substitution of a threonin for the alanin at position 6 of the nonameric epitope. Multimers were made for both epitopes since they had high affinity to HLA-A2, however only the well known WT-1 epitope yielded an actual T cell response (0.55%) in patient UPN655 who was diagnosed with AML. Another epitope to which a high number of CD8^+^ T cells were observed was IQLPHLKTL derived from the gene TLR10, which could not be confirmed by genotyping of the respective recipient-donor pair. Interestingly, SNP rs11096955 can also lead to the formation of the LQLPHLKTL epitope, which proved to have low affinity for HLA-A2 and was discarded for further experiments. However, the detected TLR10 peptide-specific T cells could be cross reactive between these two epitopes since the alternative amino acid is not present at the anchor side to the HLA molecule. Though several specific responses were detected in the screening of the enriched patient PBMC, none of these responses could be validated by conventional tetramer staining or culture methods. Known HLA-A2-restricted MiHA (i.e. HA-1, HA-2, HA-8 and SMCY) were added to the screening panel. However, we only found 1 response for the control MiHA SMCY.A2 (Table 3). DNA of all patients and their corresponding donors used in the screening was genotyped for the control MiHA. Mismatches for these MiHA were detected in 6 out of 17 screened patients. Conventional tetramer staining of the PBMC of these patients followed by tetramer validation after stimulation with peptide pulsed EBV-LCL revealed MiHA T cell responses in 4 out of the 6 mismatched patients of which 3 were not found by screening with the combinatorial encoding set.

A potential benefit of our approach is that the epitopes were predicted from a set of genes with a restricted expression pattern, which gives an advantage when looking for tissue restricted MiHA. However, there are critical limitations of our algorithmic model and selection criteria for novel candidate MiHA. While the majority of MiHA identified thus far are derived from allelic SNPs that generate an amino acid disparity (i.e, nonsynonymous SNPs) between donor and recipient, other variables may mask the presence of MiHA or otherwise confound such analyses. Many MiHA are likely to be encoded by polymorphic cDNAs or ESTs that are simply not yet represented in GenBank as translated polypeptides, such as protein isoforms derived from relatively obscure alternative splicing variants [18], cryptic translation products [19]–[21], or perhaps transposable elements (A.G.B., manuscript in preparation). Another possibility is that even though a MiHA-encoding gene is represented in GenBank, its relevant polymorphisms may not yet be fully characterized. Further complicating the algorithmic prediction of MiHA peptides is the demonstration that in certain instances, peptides can be excised and spliced in the proteasome prior to presentation by MHC [22], [23]. Another important caveat is that the selection of T cells specific for candidate antigens with MHC-multimer methodologies or T cell stimulation assays (i.e., ELISA, ELISPOT) with exogenously pulsed candidate antigenic peptides based on cDNA sequence occurs in the absence of knowledge of post-translational modifications, which can be critical to determination of immunogenicity [24]. Should reverse immunology approaches be further employed for the discovery of MiHA relevant to the immunotherapy of hematologic malignancies, it will be essential that discovery efforts are focused on those candidate MiHA polymorphisms with allele frequencies that ensure frequent donor/recipient disparity. Although progress has been made over the years to optimize epitope prediction by reversed immunology, there a still some hurdles to take before this can be implemented into a, preferably high throughput method to identify new clinical relevant MiHA. The concurrent detection of multiple epitope-specific CTL as described in this manuscript is a step forward in this process but the identification of novel MiHA by more conventional approaches should still be taken into consideration. These approaches make use of CTL clones generated from post-SCT recipients and recently genome-wide association study (GWAS) has been used for identification of the epitope recognized by these CTL ([25], Broen et al. submitted for publication). The recent addition of several new MiHA identified by GWAS shows the high feasibility of this approach. Nevertheless also this approach still is laborious as well as uncertain since clinical relevance of the discovered MiHA can only be studied in great detail after the identification has finished.

In conclusion, this study describes the use of combinatorial encoding MHC-multimers for the concurrent detection of MiHA-specific T cells after SCT and DLI. Feasibility of this approach was demonstrated by applying five-color encoded MHC multimers for a set of 10 known MiHA. In addition, the amount of patient material required is far less than for conventional, single tetramer screening. In addition, we determined the potential value of combinatorial encoding MHC multimers in MiHA identification. A set of 75 candidate MiHA peptides was predicted from polymorphic genes with a hematopoietic expression profile. Monomer-enriched patient PBMC were screened for candidate MiHA and several epitope-specific T cells were detected during the screening. Although none of these epitope-specific T cells could be confirmed by conventional techniques, the concurrent detection of MiHA specific CD8^+^ T cells by combinatorial encoding shows potential as a tool for the detection of multiple T cell responses.

## Materials and Methods

### Patient material

All patients in this study were admitted in our transplantation program from May 1996 onwards. Patients underwent HLA identical allogeneic SCT using a T cell-depleted stem cell graft containing a fixed number of 0.5×10^6^ T cells/kg body weight. Several patients also underwent subsequent prophylactic or therapeutic DLI. Peripheral blood samples of patients were collected after written informed consent in ongoing clinical stem cell transplantation protocols approved by the Radboud University Nijmegen Medical Centre (RUNMC) Institutional Review Board.

### Cell isolation and culture

CD8^+^ T cell bulk cultures were generated from peripheral blood mononuclear cells (PBMC) obtained after SCT and cultured in Iscove's modified Dulbecco's medium (IMDM; Invitrogen, Carlsbad, CA) supplemented with 10% human serum (HS; Sanquin blood bank, Nijmegen, the Netherlands). After initial stimulation, 100 IU/ml IL-2 (Chiron, Emeryville, CA) and 10 ng/ml IL-15 (BD) were added at day 2. Flow cytometric analysis was performed at day 7. All Epstein-Barr virus lymphoblastoid cell lines (EBV-LCL) were cultured in IMDM/10% fetal calf serum (FCS; Integro, Zaandam, The Netherlands).

### SNP genotyping assays

Genotyping of HLA-matched SCT donor-recipient pairs for known MiHA was conducted using the KASPar assay, a fluorescence-based competitive allele-specific PCR that utilizes non-labeled primers (KBioscience, Herts, UK). Details of the process and primer sequence can be obtained from KBioscience. SNP genotyping for verification of immunogenic epitopes was performed by Taqman SNP genotyping according to manufacturer's recommendation (Applied Biosystems [ABI], Foster City, CA). Briefly, 10 ng of gDNA was amplified using ABI pre-designed SNP genotyping assays. 5 ul of gDNA (2 ng/µl) was combined with 12.5 µl of TaqMan Universal PCR Master Mix (2×) and 1.25 µl appropriate genotyping assay reagent containing specific forward and reverse primers and specific FAM/VIC probes (20×) in a total reaction volume of 25 µl. Reactions were run on the ABI 9700 thermal cycler in a 96 well format for 1 cycle at 95°C×10 minutes followed by 40 cycles at 92°C×15 seconds, 60°C×1 minute. A post-PCR plate read for FAM/VIC was performed on the ABI 9700HT and genotype calls were generated using SDS 2.3 software (ABI).

### Prediction of HLA-A2-restricted candidate MiHA

To predict HLA-A2-restricted MiHA within hematopoiesis restricted gene products, adaptable software was written in the Java language. This software utilizes the Entrez retrieval system (http://www.ncbi.nlm.nih.gov/entrez) within the NCBI's GenBank sequence repository to extract from a database of all known human amino acid sequences (ftp://ftp.ncbi.nlm.nih.gov/genomes/H_sapiens/protein) the accession numbers of amino acid variations derived from allelic non-synonymous SNPs, as represented in GenBank's dbSNP database. The software algorithm then parses the retrieved files of polymorphisms and implements the predictive HLA-binding algorithm described by Parker et al. [26] on peptides from these files that encompass the polymorphism within a nine- or ten-mer offset window, using HLA-A2-specific coefficient files from the BIMAS website (http://www-bimas.cit.nih.gov/molbio/hla_bind/ search_info.html). Algorithmic input of gene expression data for hematopoiesis biased genes included, but were not limited to, the following resources: downloadable gene expression data sets from the Whitehead Institute for Genomics Research pertaining to expression differences between ALL and AML [27], genes upregulated in ALLs carrying a chromosomal translocation involving the mixed-lineage leukemia gene (MLL) (http://www-genome.wi.mit.edu/cgi-bin/cancer/datasets.cgi); diffuse large B-cell lymphoma gene expression profiling data sets from the Lymphoma/Leukemia Molecular Profiling Project website (http://llmpp.nih.gov/); a hematopoietic stem cell database at the Stem Cell Database website (http://stemcell.mssm.edu/v2/); and the human HapMap project at http://www.hapmap.org. In addition, manually retrieved accession numbers of hematopoietically biased genes described in the current literature represented a major source of input data for the algorithm.

### Generation of peptide-MHC multimers

Peptide-MHC multimers were produced by UV-mediated ligand exchange as previously described by Rodenko et al. [9]. The ligand used for the construction of MHC class I was a UV-sensitive peptide containing a (2-nitro)phenylglycine residue which is cleaved upon UV irradiation, making the MHC molecule peptide-receptive and capable of being loaded with epitopes of choice. The expression, purification and refolding of MHC heavy and light chains were performed as described by Gorboczi et al. [28].

### Peptide-HLA-A2-based affinity ELISA

Affinity of the predicted MiHA epitopes to HLA-A2 was tested by an HLA-A2 affinity streptavidin-based sandwich ELISA described by Rodenko et al. [9]. Briefly, 96-well plates were coated with streptavidin to which biotinylated monomers, containing all different predicted peptides, were added. Next, an HRP-conjugated anti-β2m antibody was added, binding to all stable MHC class I molecules. 2,2′-azino-bis(3-ethylbenzthiazoline-6-sulphonic acid) was added and the green-colored oxidation product was measured at OD 414 nm.

### Enrichment of peptide-specific T cells

PBMCs were obtained from CMV^+^ and EBV^+^ buffy coats (Sanquin blood bank, Nijmegen, the Netherlands) or from patients after SCT or DLI. All enrichments were performed on cryopreserved material. PBMC were stained with PE-labeled MHC multimers (1 µl of each individual PE-multimer for 10^7^ PBMC) for 1 hour at 4°C. In case of the CMV/EBV enrichment a total of 2 µl PE-multimer for each specific epitope was added, while in case of enrichment for the candidate MiHA panel a 79 µl PE-multimer mix consisting of 1 µl of each candidate and control MiHA epitope was added. Subsequently, cells were washed twice, and incubated with 20 µl magnetic beads coated with anti-PE antibody (Miltenyi Biotec, Bergisch Gladbach, Germany). Magnetically labeled cells were isolated by MACS (Miltenyi Biotech) using a MS Column following the manufacturer's protocol. Eluted cells were washed and resuspended in IMDM supplemented with 10% human serum, 100 IU/ml IL-2 and 10 ng/ml IL-15 (BD). On day 1, 2×10^4^ irradiated (60 Gy) autologous feeder cells were added along with 2,500 CD2/CD3/CD28 loaded anti-Biotin MACSiBead Particles (Miltenyi Biotec). On day 8, to prevent overgrowth by CD4^+^ T cells, T-cell cultures were depleted of residual CD4^+^ T cells via the addition of 50 µl of BD IMag anti-human CD4 particles (BD Biosciences) per 10^7^ total cells and treatment per manufacturers' protocol. Cultures were split and refreshed biweekly with medium plus cytokines. After 2–3 weeks, cell cultures were counted and tested for the presence of peptide-specific CD8^+^ T cells using combinatorial MHC multimer staining.

### Combinatorial MHC multimer staining

Combinatorial MHC multimer stainings were performed on either thawed PBMC samples or *ex vivo* expanded T cell cultures. For this, 1×10^6^ PBMCs or 1–5×10^5^ cultured T cells were incubated with 1 µl of every single MHC multimer (final concentration 2 µg/ml per MHC based on initial monomer concentration) in a total volume of 80 µl for 15 min at room temperature in the dark. Subsequently, an antibody mixture consisting of 0.8 µl CD8-Alexa700 (Invitrogen, Carlsbad, CA), 4 µl CD4-FITC (Beckman Coulter), 2 µl CD14-FITC (BD), 2 µl CD19-FITC (Dako), 6 µl CD16-FITC (BD) was added, and cells were incubated for 30 min at 4°C. Finally, cells were washed twice and Sytox Blue (Invitrogen) was added at a final dilution of 1∶5000 to allow dead cell exclusion. Data acquisition was performed on a Cyan-ADP analyzer (Beckman Coulter) and analyzed by Kaluza 1.1 software (Beckman Coulter). Antigen-specific T cells were quantified using an identical gating strategy as described by Hadrup et al. [7] and Velthuis et al [11]. First, viable Sytox Blue-negative, single-cell lymphocytes were gated. Subsequently, CD8-positive and FITC (CD4, CD14, CD16, CD19) negative cells were selected. MHC multimer positive cells were gated as CD8^+^ T cells positive in two MHC multimer channels (APC, PE, Qdot605, Qdot655, Qdot705 and Qdot800) and negative in the four other MHC multimer channels. In order to validate dual-color coded CD8^+^ T cells, PBMC were cultured in IMDM 10% HS and stimulated with irradiated EBV-LCL expressing the appropriate HLA restriction molecule. After initial stimulation, 100 IU/ml IL-2 (Chiron) and 10 ng/ml IL-15 (BD) was added at day 2. Primary T cell cultures were analyzed by flow cytometry at day 7.

## Supporting Information

Table S1 Screening pool of high and intermediate binding HLA-A2 peptides set up with combinations of labeled MHC multimers.
(DOCX)
