# Supplementary material for: Concurrent Detection of Circulating Minor Histocompatibility Antigen-Specific CD8+ T Cells in SCT Recipients by Combinatorial Encoding MHC Multimers
Source: PLoS One. 2011 Jun 24;6(6):e21266. doi: 10.1371/journal.pone.0021266 (PMC3123304; doi:10.1371/journal.pone.0021266)
Supplement: Table S1 — Screening pool of high and intermediate binding HLA-A2 peptides set up with combinations of labeled MHC multimers. (DOCX) [file pone.0021266.s001.docx]

**Supplemental Table S1.** Screening pool of high and intermediate binding HLA-A2 peptides set up with combinations of labeled MHC multimers

|  | Predicted allelic peptides | HLA-A2 binding capacity | Fluorochrome code | |
| --- | --- | --- | --- | --- |
| Pool A |  |  |  |  |
| 1 | YLATLTWML | High | PE | APC |
| 2 | YLATFTWML | High | PE | Q605 |
| 3 | LLLGRLTGVI | High | PE | Q655 |
| 5 | KLNDVLLIQL | High | PE | Q705 |
| 6 | KLNDILLIQL | High | PE | Q800 |
| 7 | LLSEAHTCV | High | APC | Q605 |
| 8 | LLSEAHTCI | High | APC | Q655 |
| 9 | RMFPNAPYL | High | APC | Q705 |
| 10 | RMFPNTPYL | High | APC | Q800 |
| 11 | KLQPYFQTL | High | Q605 | Q655 |
| 13 | RLFQESFNM | High | Q605 | Q705 |
| 16 | LAQDYLQYV | High | Q605 | Q800 |
| 22 | VSYKSFTFV | High | Q655 | Q705 |
| 27 | FLTLGVLSL | High | Q655 | Q800 |
| HA-1 | VLHDDLLEA |  | Q705 | Q800 |
| Pool B |  |  |  |  |
| 29 | ILGVLSLLL | High | PE | APC |
| 30 | TLGVLSLLL | High | PE | Q605 |
| 32 | LTLGVLSLL | High | PE | Q655 |
| 35 | ILPKMGIQNV | High | PE | Q705 |
| 37 | TLIKWSHSL | High | PE | Q800 |
| 47 | LMNDFYPGI | High | APC | Q605 |
| 48 | LMNDFYLGI | High | APC | Q655 |
| 49 | YMPQNFYCI | High | APC | Q705 |
| 50 | YMPQNFYCV | High | APC | Q800 |
| 51 | FLLEFLLPL | High | Q605 | Q655 |
| 52 | FLLEFFLPL | High | Q605 | Q705 |
| 53 | AMFLLEFLL | High | Q605 | Q800 |
| 54 | AMFLLEFFL | High | Q655 | Q705 |
| HA-2 | YIGEVLSV |  | Q655 | Q800 |
| 55 | FLLPLGIIL | High | Q705 | Q800 |
| Pool C |  |  |  |  |
| 57 | FLCKFVFTI | High | PE | APC |
| 58 | FLCKFLFTI | High | PE | Q605 |
| 67 | KLSDTLHSL | High | PE | Q655 |
| 68 | RLSDTLHSL | High | PE | Q705 |
| 75 | LQVDQLWDV | High | PE | Q800 |
| 76 | LQVDQLWDA | High | APC | Q605 |
| 81 | ILATLPEFVV | High | APC | Q655 |
| 82 | ILATLPEYVV | High | APC | Q705 |
| 83 | ILATLPEFV | High | APC | Q800 |
| 84 | ILATLPEYV | High | Q605 | Q655 |
| 95 | VLFLSVNYL | High | Q605 | Q705 |
| 96 | FLVRDEVTV | High | Q605 | Q800 |
| HA-8 | RTLDKVLEV |  | Q655 | Q705 |
| 100 | YLVPSDALL | High | Q655 | Q800 |
| 101 | LQHHEQWLV | High | Q705 | Q800 |
| Pool D |  |  |  |  |
| 4 | LLLGGLTGVI | Intermediate | PE | APC |
| 14 | RIFQESFNM | Intermediate | PE | Q605 |
| 15 | LAQDYLQCV | Intermediate | PE | Q655 |
| 17 | KMHLAHSTL | Intermediate | PE | Q705 |
| 18 | KMHLAHSNL | Intermediate | PE | Q800 |
| 21 | VSYKSLTFV | Intermediate | APC | Q605 |
| 28 | FLILGVLSL | Intermediate | APC | Q655 |
| 31 | LILGVLSLL | Intermediate | APC | Q705 |
| 36 | ILPKMGIQNA | Intermediate | APC | Q800 |
| 39 | LVGEQVTVHV | Intermediate | Q605 | Q655 |
| 41 | QILQGLGFNL | Intermediate | Q605 | Q705 |
| SMCY.A2 | FIDSYICQV |  | Q605 | Q800 |
| 90 | ALQEAMEQL | Intermediate | Q655 | Q705 |
| 44 | CLAKNSSIYV | Intermediate | Q655 | Q800 |
| 45 | LLVWYCREFA | Intermediate | Q705 | Q800 |
| Pool E |  |  |  |  |
| 46 | LLVWYCRDFA | Intermediate | PE | APC |
| 61 | LLLDPPWST | Intermediate | PE | Q605 |
| 62 | LLLNPPWST | Intermediate | PE | Q655 |
| 63 | VLLLDPPWST | Intermediate | PE | Q705 |
| 64 | VLLLNPPWST | Intermediate | PE | Q800 |
| 65 | LLLDPPWSTA | Intermediate | APC | Q605 |
| 66 | LLLNPPWSTA | Intermediate | APC | Q655 |
| 91 | FLVKCFDKT | Intermediate | APC | Q705 |
| 72 | VLTEPHLRLL | Intermediate | APC | Q800 |
| 97 | MVLEHPARV | Intermediate | Q605 | Q655 |
| 102 | SLMGILLRI | Intermediate | Q605 | Q705 |
| 85 | KLCSFQEEMV | Intermediate | Q605 | Q800 |
| 86 | KLCSFQEEMA | Intermediate | Q655 | Q705 |
| 87 | KNWTERWFV | Intermediate | Q655 | Q800 |
| 89 | ILLDENCCV | Intermediate | Q705 | Q800 |
| Pool F |  |  |  |  |
| 43 | CLAKNNSIYV | Intermediate | Q655 | Q705 |
| 71 | VLTEPQLRLL | Intermediate | APC | Q705 |
| 78 | TLTEIVRFRC | Intermediate | Q605 | Q655 |
| 79 | IQLPHLKTL | Intermediate | Q605 | Q705 |
